# Low Bone Mass and Recurrent Fractures in Neurofibromatosis With Concomitant Hemoglobin SC Disease

**DOI:** 10.7759/cureus.37868

**Published:** 2023-04-20

**Authors:** Hussam Alkaissi, Beisi Ji, Parima Saxena, Emily Kim, Navid Salahi, John Muthu, Samy I. McFarlane

**Affiliations:** 1 Internal Medicine, Kings County Hospital Center, Brooklyn, USA; 2 Internal Medicine, Veterans Affairs Medical Center, Brooklyn, USA; 3 Internal Medicine, State University of New York Downstate Medical Center, Brooklyn, USA; 4 Pathology, State University of New York Downstate Medical Center, Brooklyn, USA; 5 Medicine, Sickle Cell Division, Kings County Hospital Center, Brooklyn, USA; 6 Internal Medicine/Endocrinology, State University of New York Downstate Health Sciences University, Brooklyn, USA

**Keywords:** genetics, recurrent fractures, osteopenia, neurofibromatosis 1, sickle cell hbsc, sickle cell disease

## Abstract

Bone disease and bone loss are common features in certain monogenic diseases such as RASopathies, including neurofibromatosis (NF). Similarly, bone complications are frequent in hemoglobinopathies, another group of Mendelian diseases. This paper reports a young patient with both NF and hemoglobin SC (HbSC) diseases who had multiple vertebral fractures with osteopenia. We also discuss the cellular and pathophysiological mechanisms underlying both diseases and the factors responsible for bone pain and low bone mass in NF and hemoglobinopathies such as HbSC. This case emphasizes the importance of careful evaluation and management of osteoporosis in patients with HbSC and NF1, as both are relatively common monogenic diseases in certain communities.

## Introduction

Sickle cell disease (SCD) is the most common genetic disorder worldwide, affecting around one in 350 African-Americans. It is caused by a point mutation in the beta-globin chain of hemoglobin (HbS), where the sixth amino acid glutamate is replaced by valine resulting in inappropriate hemoglobin folding during physiological stress. Hemoglobin C (HbC), on the other hand, is a result of glutamate to lysine point mutations, resulting in similar hemoglobin crystallization yet much milder than HbS. Under settings of hypoxia, hyperosmolar states, infection, and acidity, HbS forms polymers of deoxygenated HbS within erythrocytes that facilitate a sickled morphology. Sickled erythrocytes drive vaso-occlusion, hemolytic anemia, increased blood viscosity, ischemia, reperfusion injury, and widespread inflammation [1]. The constant state of cellular injury and inflammation predisposes to multiple osseous complications, including low bone mass (osteopenia or osteoporosis), osteomyelitis, and bone infarction [2]. Neurofibromatosis type 1 (NF1) is another common genetic disorder affecting around one in 2600-3000 individuals. It is an autosomal dominant disorder caused by loss-of-function mutations of the NF1 gene. NF1 impacts virtually every organ system and can cause dermatologic, neurologic, gastrointestinal, endocrine, pulmonary, and musculoskeletal complications [3]. NF1 has profound implications on bone metabolism, manifesting as short stature, sphenoid wing dysplasia, pseudo-arthrosis of long bones, reduced bone mineral density (BMD), and scoliosis [4]. Due to the high incidence of both genetic diseases, it is possible that certain individuals might inherit both, resulting in a "double-hit" effect on certain end organs, such as the skeletal system.

In this report, we describe a rare association between NF1 and SCD in an adult patient with osteoporosis, a relationship not previously described in the literature. We discuss the independent pathophysiological mechanisms of osteoporosis in both conditions and the important therapeutic strategies needed to manage osteoporosis manifesting from a dual etiology.

## Case presentation

A 29-year-old woman with NF1, scoliosis, and hemoglobin SC (HbSC) disease was referred for evaluation of metabolic bone disease, given a history of multiple thoracic compression vertebral fractures. She has a normal menstrual cycle with no history of smoking or alcohol use.

The patient was also diagnosed with HbSC disease in childhood. She had a few episodes of acute chest syndrome and numerous hospitalizations for vaso-occlusive crisis. The disease was also complicated by avascular necrosis of the femoral head and left humeral head. The patient was diagnosed with NF1 at the age of 16 with a family history significant for NF1 in two siblings, but no history of fractures or osteoporosis. The patient has been taking daily calcium 600mg-vitamin D3 400 units tablet and has an etonogestrel implant in her left upper arm. She is also on daily folic acid 1 mg and hydroxyurea 1000 mg.

**Figure 1:**
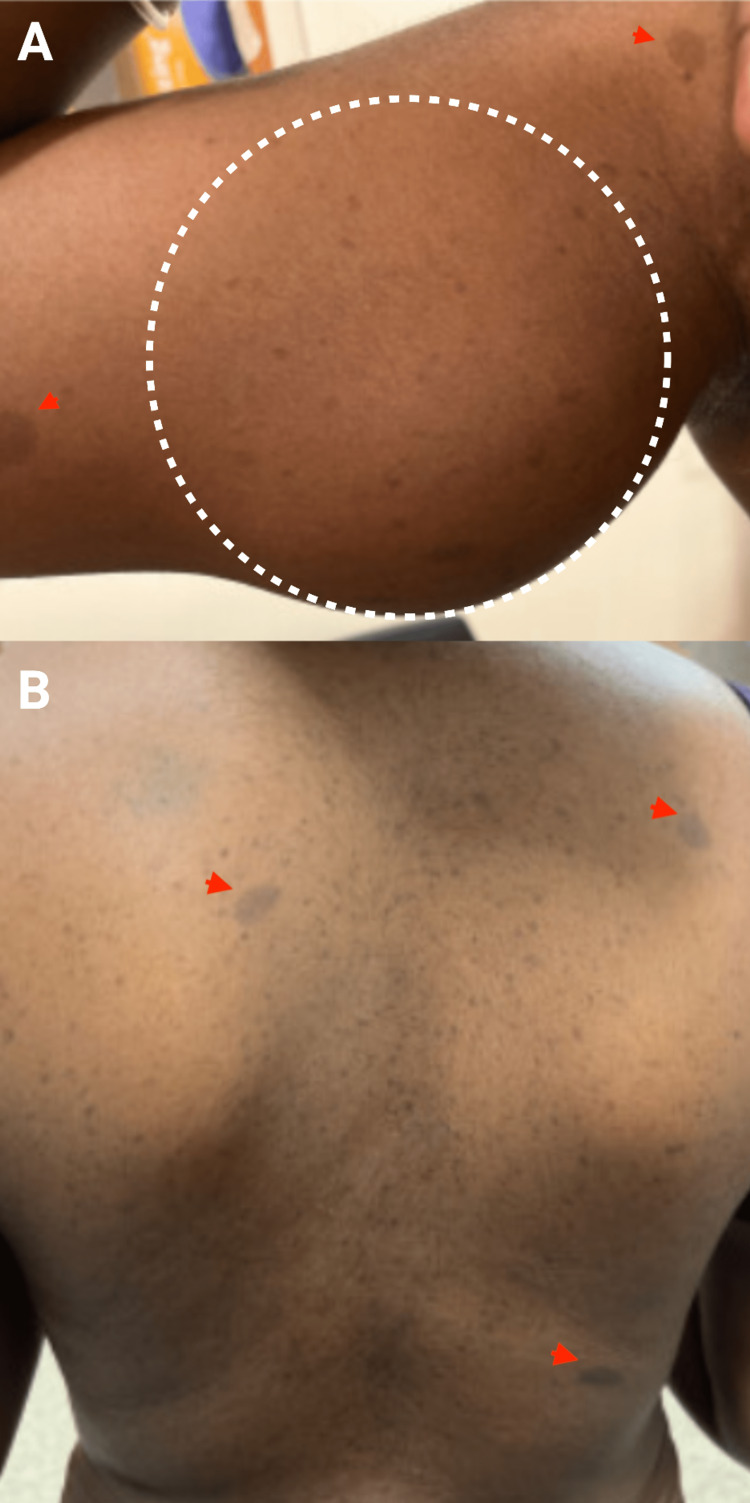
(A) A mass in the right arm on the upper medial aspect(outlined), with café-au-lait macules (arrow heads). (B) Multiple café-au-lait macules and neurofibromas on the back (arrow heads).

On examination, she had a body mass index (BMI) of 22.6 kg/m^2^ with multiple tumors in various body areas, including the right arm, right axillary, right elbow, left forehead, left intercostal muscles, and right lumbar region, with the largest tumor on her right upper arm proximal to mid humerus around 9 x 6 cm. Axillary freckling and multiple café-au-lait macules are also present (Figure 1).

**Figure 2:**
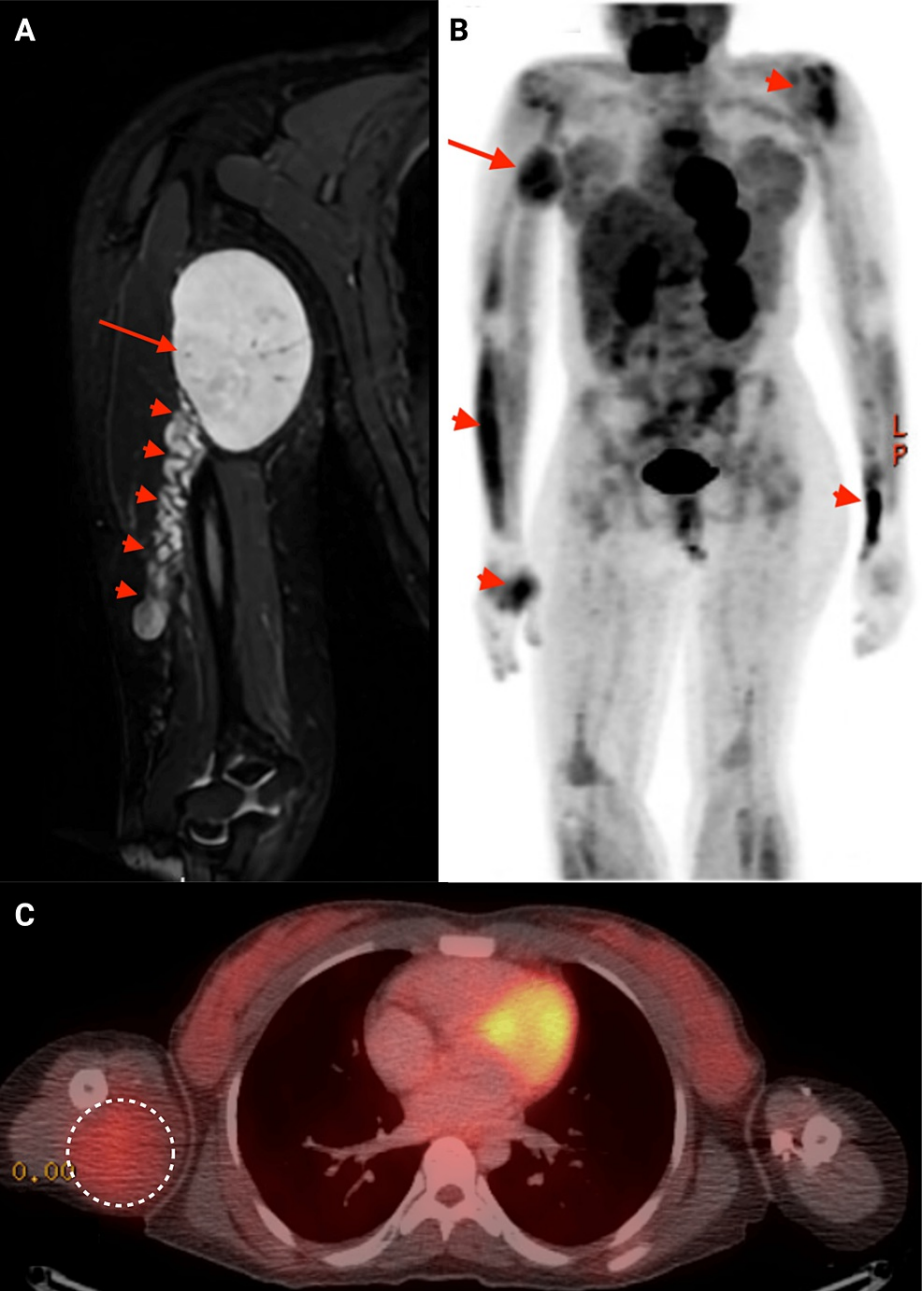
(A) Short tau inversion recovery (STIR) sequence magnetic resonance imaging (MRI) of the right upper extremity showing a large oval hyperintense mass (arrow) originating from the radial nerve, obstructing venous return due to the mass effect, with distal varicose veins (arrow heads). The mass shows increased uptake of 18-fluorodeoxyglucose (FDG) tracer in positron-emission tomography (PET) scan (B, arrow) and PET-CT (C, outlined). Several smaller masses with increased FDG uptake seen in upper extremities (B, arrow heads).

**Figure 3:**
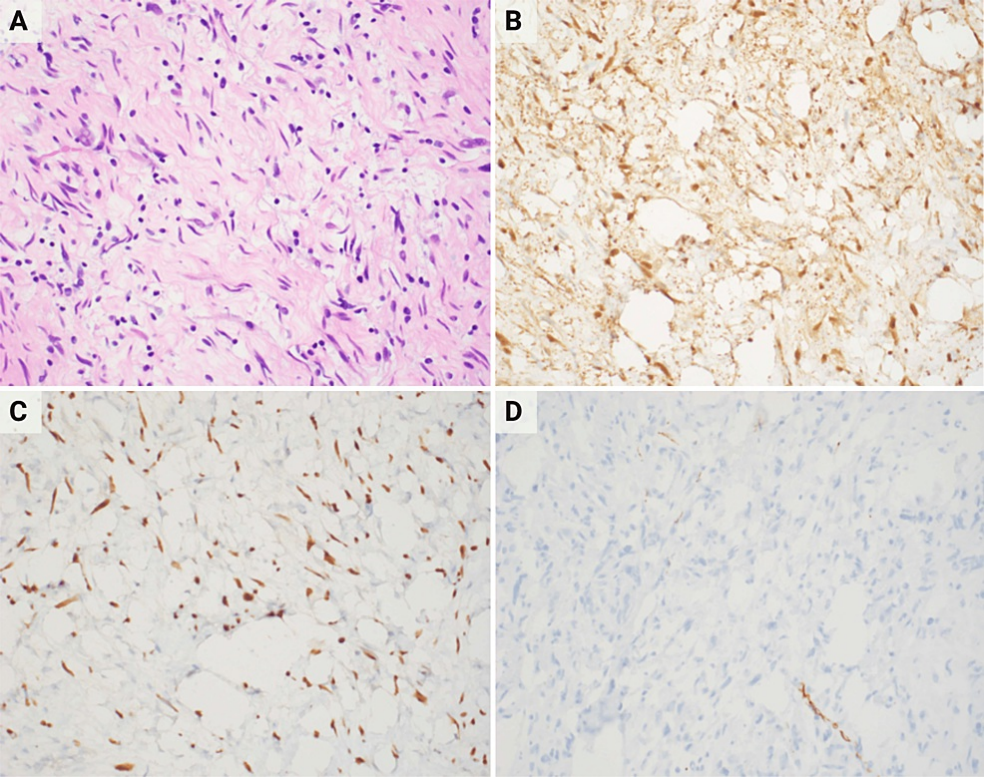
(A) Histological exam of the mass shows hypocellular loosely arranged spindle cells (H&E, 400×). Neoplastic cells are positive for S-100 (B) and SOX10 (C) on immunohistochemistry. (D) NFP immunostaining highlighting the neurofilaments. H&E: hematoxylin and eosin, NFP: neurofilament protein

A right humeral MRI was ordered to evaluate the humeral mass. It demonstrated an encapsulated mass of 8.2 x 6.1x 4.9 cm within the soft tissue of the arm medial to the humeral midshaft connected to the radial nerve. It caused distal varicose veins due to the mass effect (Figure 2A). Whole body positron emission tomography (PET) scan with an 18-fluorodeoxyglucose (FDG) tracer revealed multiple lesions, including a 5.7 x 5.6 hypodense lesions in the proximal right medial humerus with trace FDG standardized uptake value of 1.82 (Figures 2B and 2C). The biopsy of the right arm mass was consistent with the neurofibroma, with no evidence of malignancy (Figure 3). 

**Figure 4:**
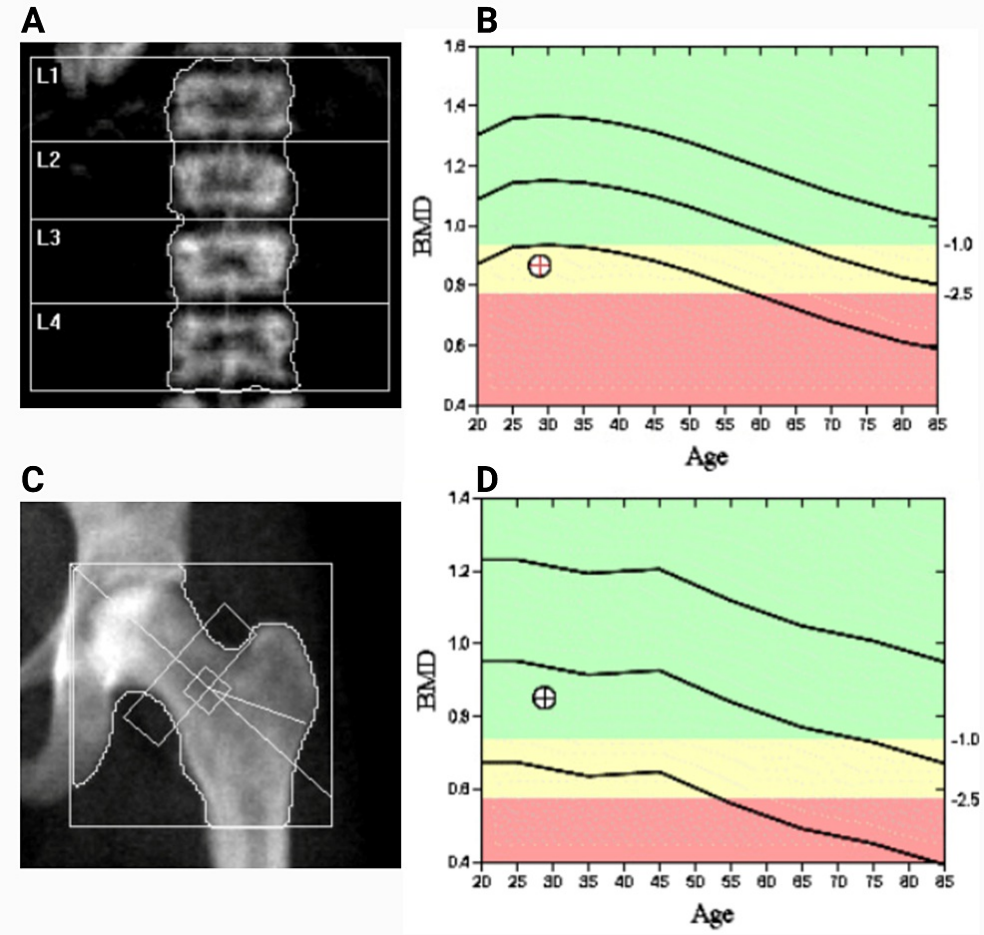
Dual-energy X-ray absorptiometry (DEXA) scan of the lumbar spine (A) with corresponding densitometry (B) and the hip and femoral neck (C) with corresponding densitometry (D) regions showing low bone mineral density as indicated by the mark on densitometry (B) lying in the yellow range. BMD: Bone mineral density

Given a history of multiple thoracic vertebral fractures, a dual-energy X-ray absorptiometry (DEXA) scan was performed which revealed a Z-score of -2.6 at the lumbar spine and -0.6 at the femoral neck, indicating low BMD (Figure 4). Serum calcium, parathyroid hormone, 25-OH Vitamin D, cortisol, and thyroid function tests were within normal limits (Table 1). She was started on zoledronic acid intravenous infusion and continued to be followed by the endocrine clinic.

**Table 1:** Routine biochemical profile, including the metabolic panel and blood count. NA: not available; WBC: white blood cell

Variable	2021	2022	2023	Reference Range
WBC (K/uL)	7.2	6.56	5.80	4.5 -10.9
Hemoglobin (g/dL)	12.5	12.1	11.5	12.0 - 16.0
Mean Corpuscular Volume (fL)	74.2	75.8	80.8	78.0 - 95.0
Platelet (K/uL)	171	143	142	130 - 400
Reticulocyte (%)	3	3.28	2.93	0.5 – 2.9
Sodium (mmol/L)	137	141	138	130 - 140
Potassium (mmol/L)	4.4	4.4	4.4	3.5 - 5.5
Alkaline Phosphatase (U/L)	57	57	55	40 - 120
Calcium (mg/dL)	9.4	9.7	9.5	8.6 - 10.0
Creatinine (mg/dL)	0.68	0.67	0.58	0.5 - 0.9
Parathyroid Hormone (pg/mL)	NA	16	NA	15 - 65
Phosphorus (mg/dL)	NA	3.0	NA	2.5 - 4.5
25-Hydroxycholecalciferol (ng/mL)	32.6	24.5	31.1	30 - 80
Thyroid-Stimulating Hormone (uIU/mL)	NA	0.41	NA	0.27 - 4.2
Cortisol AM ( ug/dL)	NA	6.4	NA	6.0 - 18.4

## Discussion

This case highlights a unique and previously unrecognized association between HbSC and NF1 in an adult patient with low bone mass. Both diseases have distinctive cellular and pathophysiological mechanisms predisposing to multiple osseous complications, including low bone mass and fractures. 

Numerous factors are responsible for bone pain and low bone density in hemoglobinopathies and result in multiple osseous complications, including low bone mass, osteomyelitis, and bone infarctions [2]. Approximately 80% of patients with hemoglobinopathies have some degree of low bone mass, with the most prominent bone loss occurring in the lumbar spine [5]. A recent study by Eskiocak et al. (2022) evaluated DEXA scans of 68 patients with SCD with a median age of 30.01 ± 8.64 years [6]. It noted that 18 (26.5%) of the 68 patients had a Z-score suggesting bone mass below the expected age-related range, while 50 (73.5%) had bone mass within the expected age range. The T-score evaluation noted that 46.8% were normal, 45.1% were osteopenic, and 8.1% were osteoporotic [6]. Multiple mechanisms can trigger bone pain and bone loss in SCD. Bone pain is usually attributed to vaso-occlusive crises and avascular necrosis, which can impair osseous healing and predispose to low BMD.

Additionally, low BMD, episodes of osteomyelitis, and osteonecrosis can all impair bone health. Chronic hemolysis also results in bone marrow expansion, associated with the widening of the medullary space [5]. Furthermore, hypoxia caused by sickling episodes can trigger erythropoietin release, which stimulates osteoclastic activity and additional bone resorption [2]. Approximately 60% of SCD patients also have severe vitamin D deficiency, worsening predisposition to low BMD [7]. Previous studies have also documented the role of primary and secondary hyperparathyroidism in bone loss in SCD [8].

Furthermore, impaired renal function and eventual development of chronic kidney disease, a late finding of SCD, drive inappropriate parathyroid hormone (PTH) secretion. Increased glomerular filtration seen in SCD can increase phosphate absorption and decrease free serum calcium, leading to increased PTH secretion by kidneys to predispose to secondary hyperparathyroidism [8,9]. Current guidelines recommend vitamin D supplementation in all deficient patients [7]. Hydroxyurea may play a role in reducing sickled RBCs, thereby preventing episodes of ischemia-reperfusion injury by reducing vaso-oclusion in trabeculae to reduce the low bone mass. However, there is a need for high-quality evidence to describe the role of hydroxyurea in osteoporosis management in SCD patients. To date, no studies have described the role of bisphosphonates in SCD patients. 

**Figure 5:**
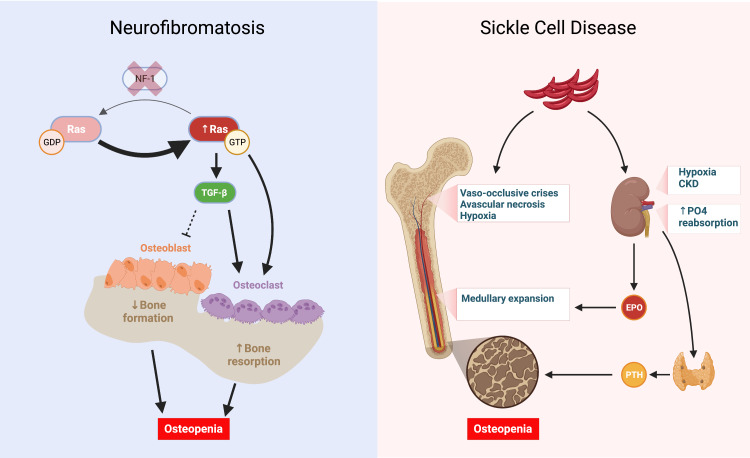
Independent pathways work synergistically to cause osteopenia and low bone density. In neurofibromatosis, the loss of NF-1 causes RAS to be stuck in an active, GTP-bound state, leading to increased osteoclastic activity. Similarly, increased RAS activity leads to increased TGF-ß, which inhibits osteoblastic differentiation and stimulates osteoclastic activity. In sickle cell disease, recurrent vaso-occlusive crises and avascular necrosis, accompanied by medullary expansion, lead to low bone density. Chronic kidney disease and increased tubular phosphate reabsorption lead to increased parathyroid hormone, further worsening osteopenia. CKD: Chronic kidney disease, EPO: Erythropoietin, GDP: Guanosine diphosphate, GTP: Guanosine triphosphate, NF1: Neurofibromin 1, PO4: Phosphate, PTH: Parathyroid hormone, Ras: Rat sarcoma virus protein, TGF-ß: Transforming growth factor ß. Created with BioRender.com

Similarly, NF1 patients have a wide range of skeletal and osseous abnormalities, including short stature, sphenoid wing dysplasia, pseudoarthrosis (failure of fracture healing) of long bones, low BMD, and scoliosis [4]. Studies have demonstrated that low BMD is prevalent in 20-50% of all NF1 patients, and like SCD, it is associated with severe vitamin D deficiency in approximately 75% of patients [10]. Seitz et al. (2010) conducted a study involving bone biopsies in 14 NF1 patients who demonstrated significantly decreased BMD compared to healthy controls. In addition, the pathology demonstrated significantly increased numbers of osteoblasts and osteoclasts, likely indicating high bone turnover. Cholecalciferol replacement in these patients resulted in a significant increase in BMD after one year of treatment [10]. To date, no precise mechanism of NF1-induced low BMD has been confirmed; however, multiple biochemical and cell signaling mechanisms are thought to influence osteoporosis development. One of the mechanisms widely supported in the literature involves intracellular Ras activity. Mouse models that mimic NF1 via gene deletion demonstrate that NF1 gene deletion results in a pathologic increase in intracellular Ras activity, triggering osteoclastic induction and inhibition of osteoblastic differentiation. In addition, osteoclasts isolated from NF1 patients had elevated Ras/PI3K activity and increased lytic activity [11]. Another theory of low BMD in NF1 involves dysregulation of the TGF-β1 signaling cascade. In vivo mouse models in NF1 knockout mice demonstrate that TGF-β1 levels are fivefold increased, associated with osteopenia, and using a TGF-β1 inhibitor, SD-208, can rescue bone marrow density (Figure 5) [12].

Interestingly, osteoclasts isolated from NF1 patients are resistant to bisphosphonate-induced apoptotic signals [13]. Furthermore, a prospective study involving seven NF1 patients demonstrated that after 23 months of alendronate therapy, there was no significant increase in bone marrow density in study subjects [14]. Current management guidelines recommend vitamin D supplementation for all NF1 patients, with no clear role for bisphosphonate use at this time [15]. Our patient is currently being managed with vitamin D, with bisphosphonates, given concomitant low BMD in the setting of HbSC disease.

One report has documented the association between SCD and NF1 in a pediatric patient, describing the development of papilledema [16]. However, the report does not describe metabolic bone abnormalities associated with both conditions. Effectively this is the first case report documenting the association of NF1 and SCD as a double-edged sword, contributing to the development of low BMD. We recommend that managing such cases involve a multifaceted approach to address the underlying pathophysiology of both diseases. Workup of low bone mass in such cases should involve investigating vitamin D deficiency and assessing parathyroid hormone, as both can be impacted in SCD and NF1. In addition, hydroxyurea can decrease vaso-oclusive crises, which is one of the contributors of bone loss in SCD.

## Conclusions

We present a case of combined NF1 and HbSC disease in a patient, demonstrating a unique association with two independent diseases predisposing to low BMD and fractures. We outlined the pathophysiology of low bone mass in each disease and described the distinctive management considerations. Finally, we proposed a management plan addressing the pathophysiologic derangements leading to low BMD in each of the two disorders. Future studies should consider investigating the role of hydroxyurea and bisphosphonates in order to identify the most effective preventive and therapeutic strategies for bone disorders in these vulnerable patient populations with genetic diseases that affect bone density, such as neurofibromatosis and sickle cell diseases. Early recognition and management of osseous complications of such disorders are of paramount importance.
